# Qualitative Analysis of the Cementation Line of Metal‐Ceramic Crowns Luted With Resin Cement: A Clinical Case Report

**DOI:** 10.1155/crid/2901390

**Published:** 2026-09-06

**Authors:** Camila Bortoletto Schoba, Milton Edson Miranda, Eveline Freitas Soares, Raquel Viana Rodrigues

**Affiliations:** ^1^ Department of Prosthodontics, School of Dentistry, Faculdade Sao Leopoldo Mandic Campinas, Campinas, State of São Paulo, Brazil; ^2^ Department of Restorative Dentistry, Dental Materials Division, Piracicaba Dental School, University of Campinas, Piracicaba, Avenida Limeira, Bairro Areiã, Brazil, unicamp.br; ^3^ Department of Oral Health Sciences, Faculty of Dentistry, University of British Columbia, Wesbrook Mall, Vancouver, Canada

**Keywords:** case report, cement excess, cementation, marginal adaptation, metal-ceramic crowns, resin cement, scanning electron microscopy

## Abstract

Unlike conventional water‐soluble luting cements, which gradually dissolve in oral fluids and partially self‐correct over time, polymerized resin cement is insoluble and will not be displaced from the restoration margin without active mechanical intervention. This case report describes the qualitative analysis of the cementation line of three metal‐ceramic crowns 1 year after placement using scanning electron microscopy (SEM) and the replica technique, and the subsequent clinical management. A 45‐year‐old female patient presented with crowns on Teeth 11, 21, and 22 cemented with a self‐adhesive dual‐cure resin cement (RelyX U200, 3 M ESPE). Despite presenting with clinical signs of localized gingivitis, the patient reported no pain or discomfort and was entirely unaware of the presence of residual cement. SEM analysis revealed cement excess of 200–500+ *μ*m, 10–23 times the manufacturer‐specified film thickness of 22 *μ*m, in cervical, interproximal, and subgingival regions. Following mechanical cement removal, resolution of bleeding on probing and reduction in probing depths were observed at all affected sites, providing direct clinical evidence of a causal relationship between residual resin cement and local gingival inflammation. These findings highlight the limitations of symptom‐based monitoring and underscore that postcementation polishing is a definitive clinical requirement, not a discretionary step.

## 1. Introduction

Prosthetic rehabilitation with metal‐ceramic crowns remains one of the most clinically established approaches in fixed prosthodontics [1]. These restorations combine the structural reliability of a metallic substructure with the esthetic qualities of dental ceramics, meeting functional and esthetic demands simultaneously [2–4]. However, long‐term treatment success depends not only on the quality of the prosthetic design and tooth preparation but critically on the management of the cementation phase.

Historically, water‐based luting cements, including zinc phosphate and conventional glass ionomer, were the standard materials for cementation of metal‐ceramic crowns [5–8]. Despite their well‐documented clinical success over decades, these materials are inherently soluble in oral fluids [5]. Their solubility, although considered a disadvantage in terms of long‐term marginal seal integrity, had an important practical consequence: residual cement excess deposited at the cementation margin during crown seating would gradually dissolve and wash away over time. Clinicians could rely on this natural washout as a secondary mechanism for managing cement excess, and cementation protocols with these materials were, in part, forgiving of imperfect intraoperative technique.

The widespread adoption of resin‐based luting cements has fundamentally changed this clinical reality [6–9]. Resin cements exhibit minimal solubility in oral fluids, superior mechanical properties, and are capable of forming both micromechanical and chemical bonds to tooth structure and restorative materials, contributing to improved marginal seal and restoration retention [9–11]. However, unlike conventional water‐soluble cements, once a resin cement has undergone polymerization, any residual excess is permanently retained at the cementation interface unless physically removed. Among resin cements, self‐adhesive formulations, such as RelyX U200 (3 M ESPE, St. Paul, Minnesota, United States), simplify the bonding protocol by eliminating the need for a separate adhesive or primer, as their phosphate‐containing methacrylate monomers interact simultaneously with tooth structure and metal oxide surfaces in a single‐step application [7, 11]. Although this simplification reduces the technical demands of the bonding step, it does not reduce the demands of postcementation management. The insolubility of these materials means that any excess remaining after cementation will not self‐correct over time, making resin cements intrinsically more technique‐sensitive with respect to cleanup than their water‐soluble predecessors [12–14].

The clinical viability of resin cements for metal‐ceramic restorations depends on the ability of phosphate‐containing functional monomers to bond to both the tooth and the metal substructure. Yoshida et al. [15] demonstrated that 10‐methacryloyloxydecyl dihydrogen phosphate (10‐MDP) forms a chemically stable bond to hydroxyapatite through its phosphate group, with a low calcium salt dissolution rate confirming the durability of this interaction at the tooth interface, whereas its methacrylate group copolymerizes with the resin matrix. The same phosphate chemistry enables interaction with metal oxide layers on alloy substructures, providing adhesion to metal that was previously difficult to achieve with water‐based cements. Additional surface conditioning protocols, including airborne‐particle abrasion and metal primer application, further strengthen the adhesive interface between resin cement and metal alloy [16–19].

Despite their clinical advantages, the insolubility and polymerization kinetics of resin cements impose a strict requirement: complete removal of cement excess is not optional but a definitive postcementation step. Residual resin cement in interproximal and subgingival regions has been associated with plaque accumulation, gingival inflammation, periodontal tissue breakdown, and secondary caries, all of which compromise long‐term restoration survival and patient periodontal health [12–14]. Yet the true extent of cement excess at the cementation interface of existing restorations is difficult to evaluate clinically.

Despite presenting with clinical signs of localized gingivitis, the patient reported no pain or discomfort and remained entirely unaware of the presence of residual cement at the cervical margins, a finding that highlights the limitations of symptom‐based clinical monitoring in detecting resin cement‐related complications. The subsequent resolution of periodontal inflammation following targeted cement removal provides direct clinical evidence, rather than inference, of a causal relationship between residual resin cement and local gingival disease. This case report describes the qualitative evaluation of the cementation line of three existing metal‐ceramic crowns 1 year after placement using SEM and the replica technique, the clinical management of identified cement excess, and the periodontal outcomes at follow‐up.

## 2. Case Presentation

### 2.1. Clinical Examination

A 45‐year‐old female patient presented for a routine dental consultation. The patient reported no relevant medical or family history and was not taking any medications at the time of examination. Clinical examination and periapical x‐rays revealed that Teeth 11, 21, and 22 had intra‐radicular cast metal posts (in good condition) and metal‐ceramic crowns placed approximately 1 year prior by a previous clinician. The previous clinician was contacted and confirmed that RelyX U200 (3 M ESPE, St. Paul, Minnesota, United States), a self‐adhesive dual‐cure resin cement, had been used for crown cementation. Metal‐ceramic restorations had been selected by the patient due to their lower cost relative to all‐ceramic alternatives and the patient′s low smile line, which did not compromise esthetic outcomes; the patient had been informed of alternative options, potential benefits, and associated risks at the time of the original treatment.

**Figure 1:**
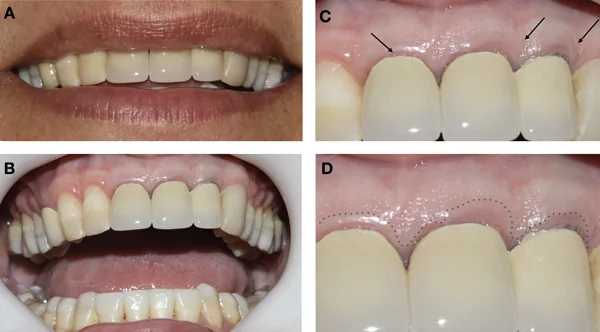
Clinical photographs at baseline examination. (A) Frontal smile view showing a low smile line that does not expose the gingival margin. (B) Close‐up of Teeth 11, 21, and 22 with metal‐ceramic crowns. (C) Signs of localized gingivitis indicated by modification of the free gingival papilla (arrows), most prominent at Teeth 21 and 22. (D) Discontinuity of the free gingival margin and the keratinized gingiva can be observed.

The patient also presented with old, discolored composite restorations exhibiting localized biofilm retention. Oral hygiene was satisfactory; however, signs of localized gingivitis were detected, with modifications of the free gingival papilla visible at Teeth 11, 21, and 22, most prominent at Teeth 21 and 22 (Figure 1). Clinical inspection of the cervical margins revealed the presence of resin cement excess that had not been removed at the time of cementation, predominantly in the interproximal and cervical regions of Teeth 11, 21, and 22, and associated with the observed gingival changes. The patient reported no pain or discomfort and was unaware of any gingival alteration. Baseline periodontal parameters, including probing depths, recession, and bleeding on probing, were recorded and are reported in Table 1. Written informed consent was obtained prior to case documentation and impression procedures (Ethics Committee, Faculdade São Leopoldo Mandic, Protocol No. 0765/2016, February 3, 2016).

**Table 1:** Periodontal parameters at baseline and follow‐up for Teeth 11, 21, and 22.

Tooth	Surface	Baseline									Follow‐up									BOP	
		PD (mm)			Rec (mm)			CAL (mm)			PD (mm)			Rec (mm)			CAL (mm)				
		M	Mid	D	M	Mid	D	M	Mid	D	M	Mid	D	M	Mid	D	M	Mid	D	Baseline	Follow‐up
**11**	Buccal	4	3	4	0	0	0	4	3	4	3	3	3	0	0	0	3	3	3	+	−
	Palatal	3	2	2	0	0	0	2	2	2	2	2	2	0	0	0	2	2	2	−	−
**21**	Buccal	4	4	3	0	0	1	4	4	4	3	3	3	0	0	1	3	3	4	+	−
	Palatal	4	2	2	0	0	0	3	2	2	3	2	2	0	0	0	3	2	2	−	−
**22**	Buccal	4	4	3	0	1	1	4	5	4	2	2	2	0	1	1	2	3	3	+	−
	Palatal	4	2	3	0	0	0	4	2	3	3	2	3	0	0	0	3	2	3	+	−

*Note:* Baseline, before cement removal; follow‐up, after cement removal.

Abbreviations: BOP, bleeding on probing (+ = present; − = absent); CAL, clinical attachment level (PD + recession); D, distal; M, mesial; Mid, mid; PD, probing depth; Rec, recession.

### 2.2. Impression Procedure

**Figure 2:**
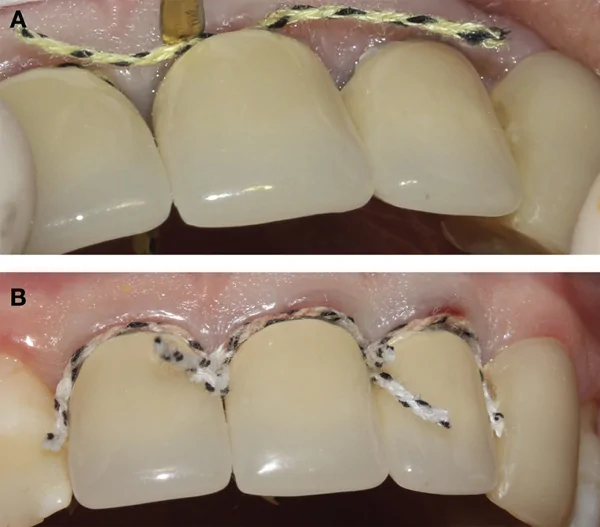
Placement of Ultrapak retraction (A) cord #000 and (B) cord #0 (Ultradent Products, South Jordan, Utah, United States) in the buccal sulcus prior to the impression procedure.

**Figure 3:**
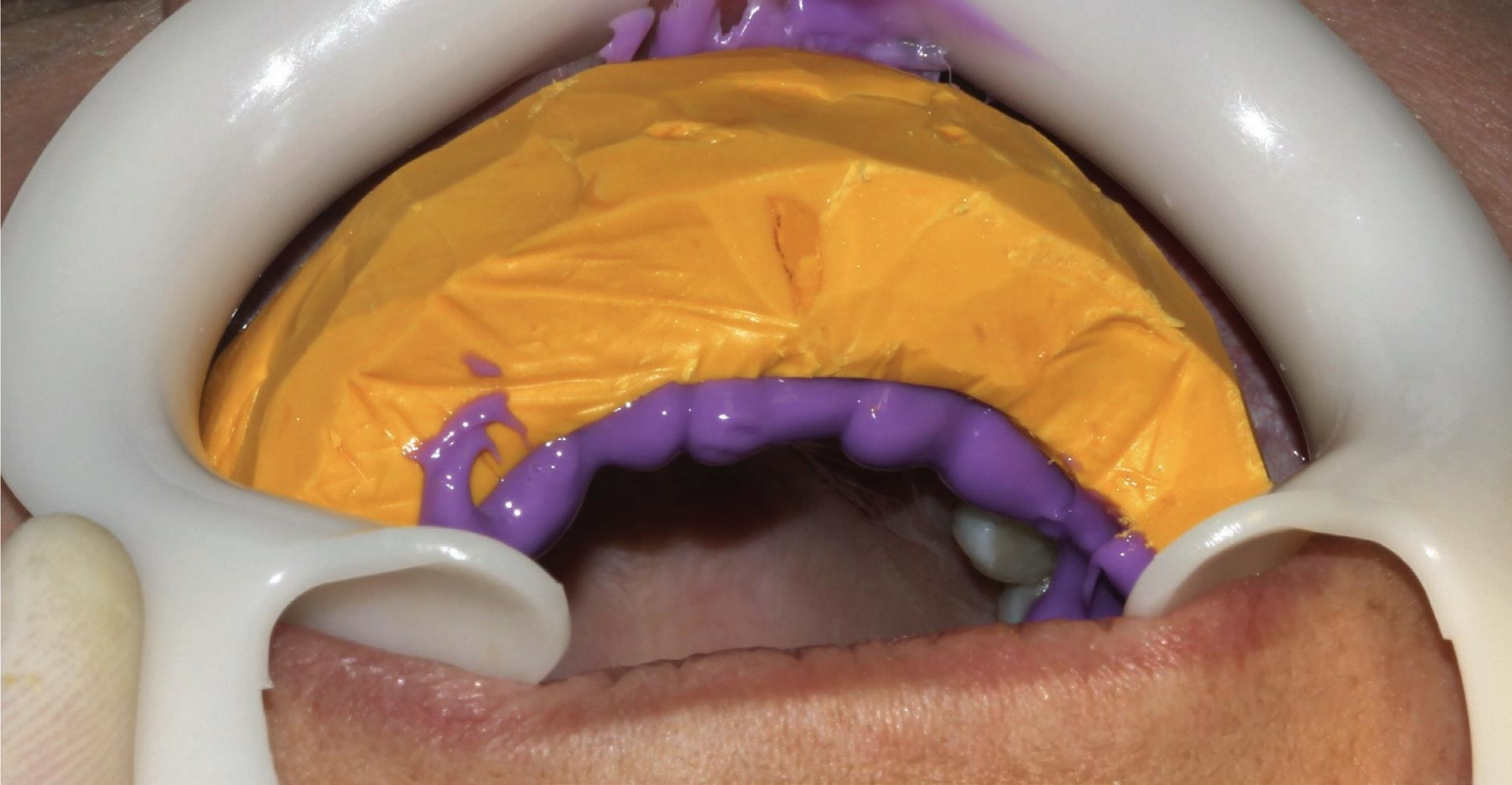
Polyvinylsiloxane impression using a two‐step putty‐wash technique (Express Standard, 3 M ESPE, St. Paul, Minnesota, United States) to capture subgingival details of the restored teeth.

To obtain accurate replicas of the clinical condition of the restored teeth, Ultrapak retraction cord #000 and #0 (Ultradent Products, South Jordan, Utah, United States) were placed in the buccal sulcus to facilitate access for the impression material (Figure 2). A two‐step putty‐wash technique was employed using polyvinylsiloxane addition silicone: heavy‐body putty in the buccal surface without a tray and light‐body material injected around the restored teeth to capture subgingival details (Express Standard, 3 M ESPE, St. Paul, Minnesota, United States) (Figure 3).

A first impression was discarded after serving to remove surface debris and contaminants, thereby improving the quality of subsequent replicas. A second impression was taken using the same technique, and the mold was filled with epoxy resin for 24 h to ensure complete polymerization. These replicas were used for SEM analysis of the cementation interface.

### 2.3. SEM Observation

Epoxy resin replicas were removed from the molds, cleaned in an ultrasonic bath with distilled water, mounted on metal stubs using double‐sided carbon tape, kept overnight in a desiccator, and sputter‐coated with gold (SCD 050 Sputter Coater, Balzers Union Aktiengesellschaft; 52 mA, 100 s). Specimens were analyzed using a scanning electron microscope (JSM/5600LV, JEOL, Tokyo, Japan; 15 kV) at the Laboratory of Electron Microscopy, Piracicaba Dental School (FOP–UNICAMP, Brazil). The entire finish line surface was evaluated, with particular attention to (1) integrity of the finish line seal; (2) presence of marginal gaps; (3) supragingival cement excess; and (4) subgingival cement excess. Cement thickness at the finish line was estimated using the calibration tool of ImageJ software (ImageJ 1.45, NIH, Bethesda, United States), with the scale bar provided in each SEM image used as the dimensional reference.

### 2.4. SEM Findings

**Figure 4:**
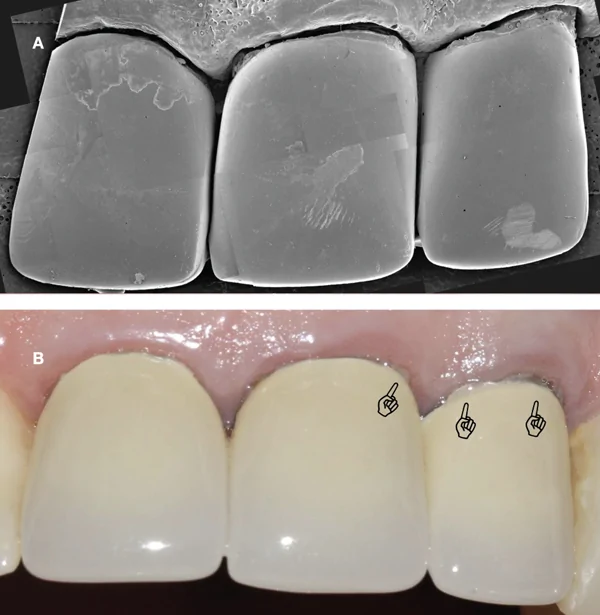
(A) Photomontage of SEM images of the entire replicated region of interest. (B) Clinical image showing visible resin cement excess in the cervical region (arrows), correlating with the SEM findings.

SEM micrographs revealed clear evidence of resin cement excess that had not been removed during or after cementation and photoactivation. No final polishing appeared to have been performed in the cervical region. Figure 4 provides a photomontage correlating the clinical and SEM findings.

**Figure 5:**
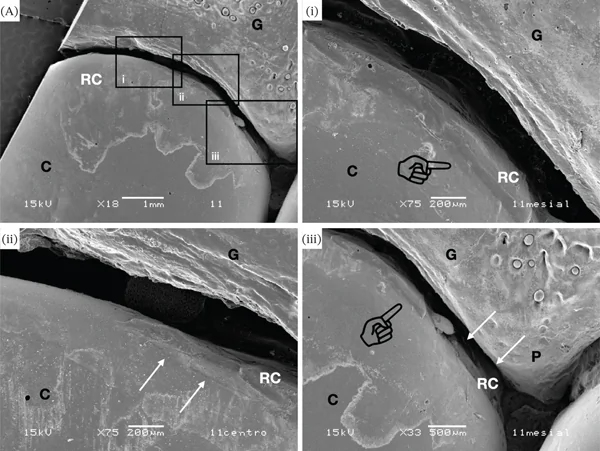
(A) SEM micrograph of the cervical finish line of Tooth 11 (18x magnification). C, crown; G, gingiva; RC, resin cement. Insets: (i) facial cement excess approximately 200 *μ*m extending subgingival (pointer); (ii) discrete facial excess with subgingival extension (arrows); (iii) interproximal area with marked subgingival cement excess (arrow) and cement attached to the buccal‐cervical area (pointer).

**Figure 6:**
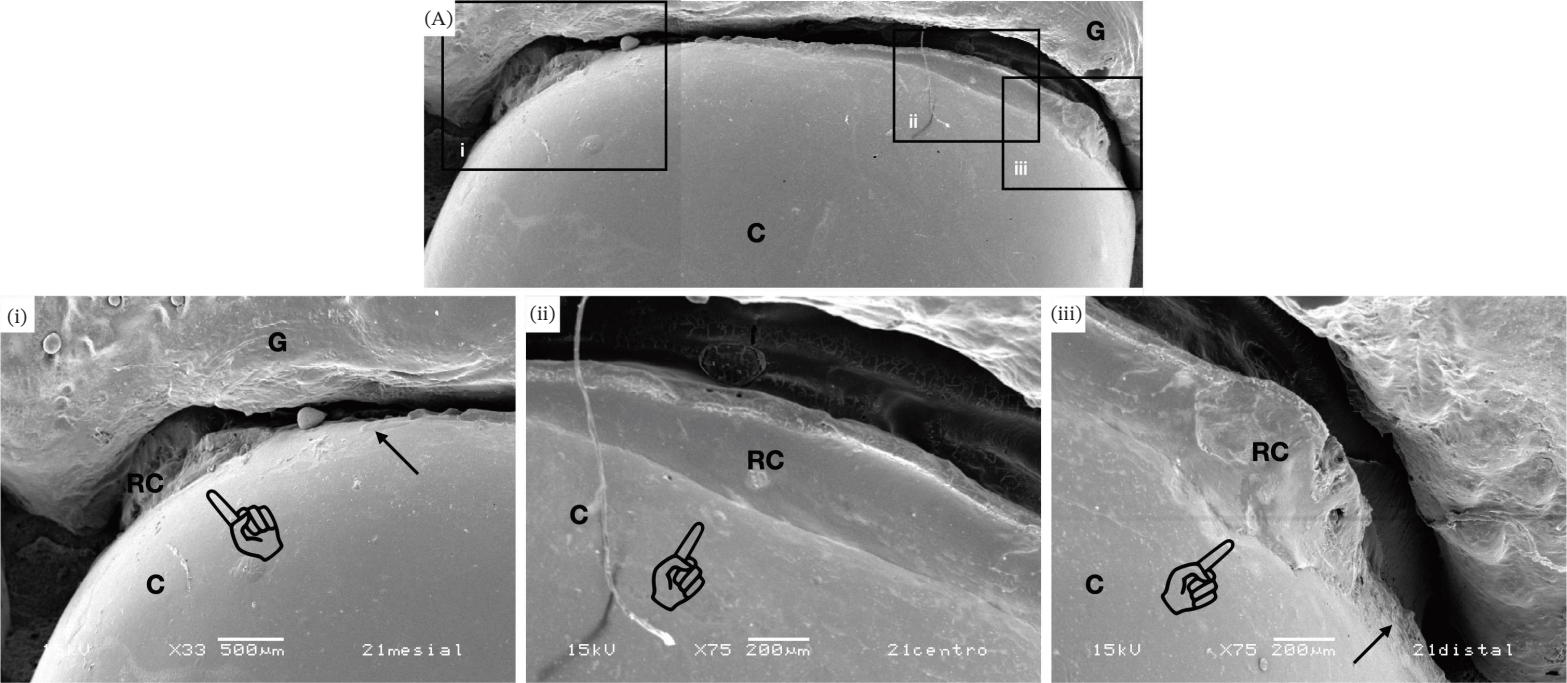
(A) SEM montage of the cervical finish line of Tooth 21. C, crown; G, gingiva; RC, resin cement. Insets: (i) mesial resin cement excess > 500 *μ*m with subgingival extension (pointer) and decreasing towards the mid area (arrow); (ii) mid‐distal excess approximately 400 *μ*m continuing subgingival (pointer); (iii) distal excess > 400 *μ*m (pointer); with subgingival extension (arrow).

**Figure 7:**
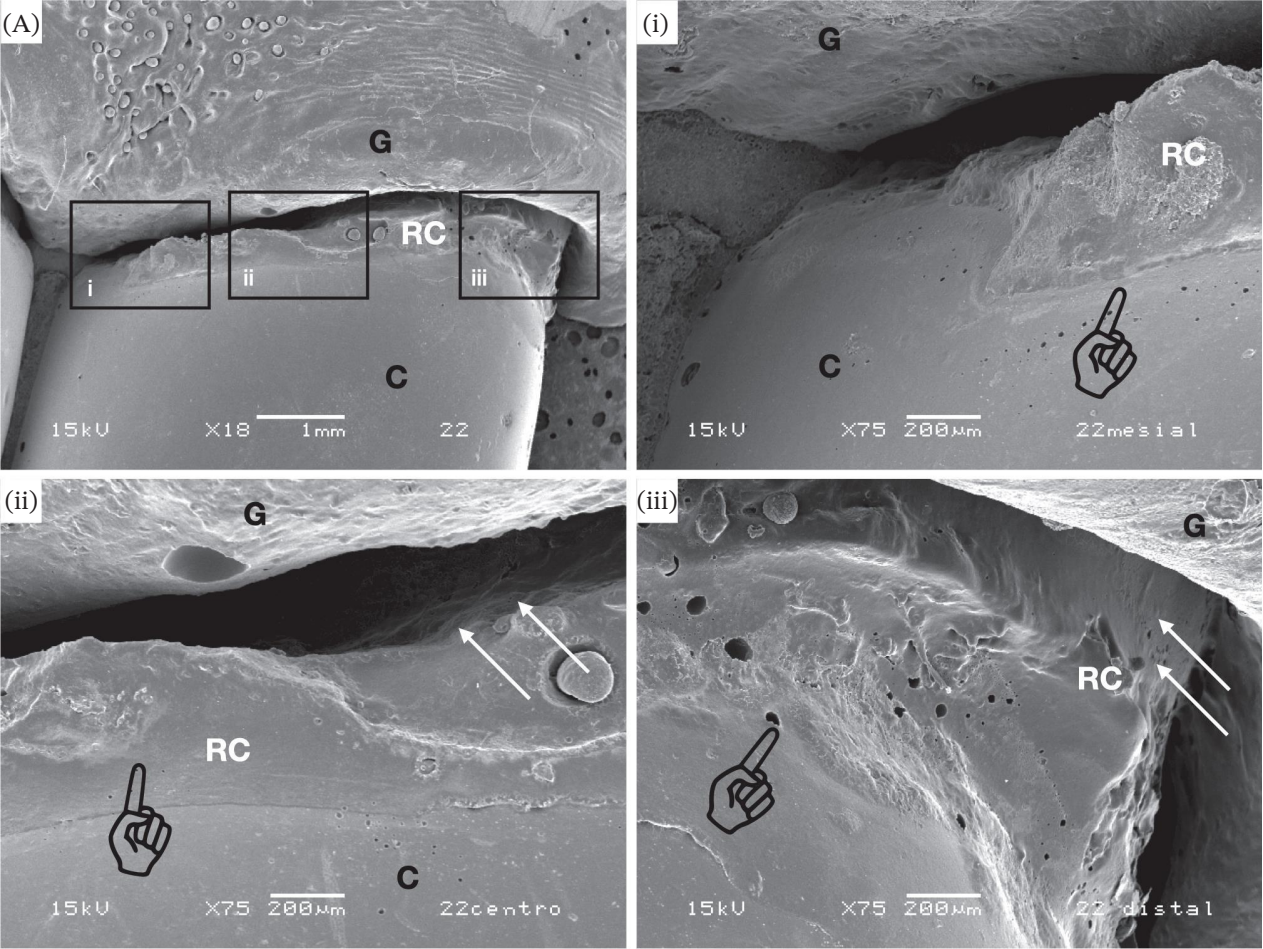
(A) SEM micrograph of Tooth 22 (18x magnification). C, crown; G, gingiva; RC, resin cement. Insets: (i) cement excess approximately 500 *μ*m with subgingival extension (pointer) in the mesial; (ii) excess approximately 500 *μ*m extending subgingivally (arrows) in the mid‐facial; (iii) distal area with cement covering crown and root surface, continuing subgingivally (pointer and arrows). Pores visible in some areas are epoxy resin artifacts.

On Tooth 11, cement excess was relatively discrete (approximately 200 *μ*m) on the facial surface, with the interproximal area showing more pronounced subgingival extension (Figure 5). On Tooth 21, resin cement excess exceeded 500 *μ*m on the mesial surface and was approximately 400 *μ*m in mid‐distal and distal regions, with visible subgingival extension (Figure 6). Tooth 22 showed similar findings, with cement excess of approximately 500 *μ*m on the mesial and mid‐facial surfaces and pronounced subgingival extension on the distal aspect (Figure 7). Pores observed in some replica areas represent epoxy resin pouring artifacts and do not reflect actual surface texture.

The patient reported no pain or discomfort associated with the crowns and was unaware of the presence of residual cement at the cervical margins. She expressed willingness to proceed with further clinical management once the findings were explained to her

### 2.5. Management of Cement Excess

At a subsequent appointment, clinical management of the identified resin cement excess was performed. Residual cement deposits were removed using a combination of hand instruments (scalers and curettes), fine finishing burs, and rubber polishing cups with a 3 M ESPE polishing system (3 M, St. Paul, Minnesota, United States). Supragingival and marginal cement was systematically removed and the surfaces were polished to restore marginal smoothness. Cement excess in the interproximal regions of Teeth 21 and 22, where values exceeded 400–500 *μ*m, was also accessed and removed. No further signs of inflammation were detected at those sites at the follow‐up assessment. The patient tolerated the procedure well, with no adverse events reported.

### 2.6. Follow‐Up Assessment

A follow‐up appointment was conducted after 4 weeks to evaluate the periodontal response following cement removal. Clinical periodontal parameters, including probing depths, recession, and bleeding on probing, were reassessed at all restored sites and are reported in Table 1. Probing depths were reduced and bleeding on probing resolved at the previously affected buccal sites of Teeth 11, 21, and 22, indicating clinical improvement of the periodontal condition following cement removal. No further signs of inflammation were detected at the interproximal regions of Teeth 21 and 22. The patient reported no pain or discomfort. No clinical photographic documentation was obtained at the follow‐up visit.

## 3. Discussion

This case report demonstrates significant resin cement excess at the cervical margins of three metal‐ceramic crowns 1 year after cementation, documented using the noninvasive SEM replica technique and quantified by ImageJ‐based image analysis [20]. The findings are clinically meaningful not only because of the magnitude of the excess observed, but because the specific material used, RelyX U200 (3 M ESPE), a self‐adhesive dual‐cure resin cement, is insoluble once polymerized, rendering spontaneous self‐correction impossible.

To fully appreciate the clinical significance of these findings, it is necessary to consider how the transition from water‐based to resin‐based luting cements has changed the management demands of crown cementation. For decades, water‐based cements, including zinc phosphate and conventional glass ionomer, were the standard materials for metal‐ceramic crown cementation [5–8]. These materials are characterized by inherent solubility in oral fluids [5, 6], which, although recognized as a limitation in terms of long‐term marginal seal integrity, provided a degree of clinical self‐correction: Residual excess cement deposited at the restoration margin during crown seating would gradually dissolve and wash away over time. This natural dissolution reduced the clinical impact of imperfect intraoperative technique and meant that postcementation finishing and polishing, although desirable, were not strictly necessary to control cement accumulation.

Contemporary resin cements fundamentally altered this paradigm. These materials exhibit minimal solubility in oral fluids and high resistance to hydrolytic and enzymatic degradation [6, 7]. Their polymerized matrix is stable, which accounts for their superior mechanical performance and marginal seal, but it also means that any residual cement excess will remain permanently at the cementation interface unless physically removed by the clinician. Finishing and polishing after resin cementation is therefore not a discretionary refinement but a definitive clinical requirement. In this context, the cementation protocol for resin cements must be more rigorous, not less, than that previously required for traditional water‐soluble materials [8, 9, 12].

This requirement is particularly important to recognize when using self‐adhesive resin cements such as RelyX U200. These formulations are designed to simplify the bonding protocol by eliminating the need for separate acid etching, priming, and adhesive application steps, making them appealing in clinical practice for their perceived ease of use [7, 11]. However, this clinical simplicity in the bonding phase does not extend to postcementation management. RelyX U200 has a manufacturer‐specified film thickness of approximately 22 *μ*m, yet the cement excess documented in this case ranged from approximately 200 *μ*m to more than 500 *μ*m, corresponding to approximately 10–23 times the designed film thickness and substantially exceeding the optimal luting cement film of approximately 25–40 *μ*m reported in the literature [21]. Furthermore, the dual‐cure mechanism of RelyX U200 initiates chemical polymerization immediately upon mixing, progressively limiting the working window during which excess cement can be easily displaced. Once the material enters the initial gel phase and begins to polymerize in the subgingival sulcus, it becomes increasingly adhesive and difficult to remove [22]. The phosphate‐containing methacrylate monomers that enable adhesion to both tooth structure and the metal oxide surface, as characterized for analogous functional monomers by Yoshida et al. [15], also contribute to the tenacious retention of excess cement on adjacent tooth and soft tissue surfaces. These material properties collectively mean that the window for successful cement removal is narrow, and any delay in cleanup has permanent consequences.

The SEM findings, confirmed by ImageJ‐based thickness measurements, document these consequences at 1 year postcementation. Cement excess was most pronounced in interproximal and subgingival regions, precisely the areas where clinical access for cement removal is most limited [22]. Mitchell et al. [23] demonstrated that adhesive cement remnants persist on tooth surfaces even after conventional cleaning attempts at the time of cementation, consistent with the present findings. Surface conditioning protocols, including airborne‐particle abrasion and metal primer application, further enhance the adhesive interface between resin cement and metal alloy [16–19]; the same surface reactivity that ensures reliable crown retention contributes to the tenacious adhesion of excess cement to surrounding structures.

Perhaps the most clinically significant finding of this case is the periodontal response observed following cement removal. At baseline, bleeding on probing was detected at the buccal surfaces of all three teeth, and increased probing depths were recorded at multiple sites, most notably at Tooth 22, where the mesial‐distal probing depth on the buccal surface measured 4–5 mm with 1 mm of gingival recession, yielding a clinical attachment level of up to 5 mm. These sites with the greatest periodontal involvement corresponded directly to those with the most pronounced cement excess on SEM. At the follow‐up assessment, bleeding on probing had resolved at all previously affected buccal sites, and probing depths were reduced, particularly at Tooth 22 where buccal depths decreased from 4–5 mm to 2–3 mm. This pattern of periodontal improvement following targeted cement removal provides clinical evidence of a causal relationship between subgingival resin cement excess and local gingival inflammation in this patient, and directly supports the biological plausibility of the association between residual resin cement and periodontal tissue breakdown documented in the literature [12–14]. Edelhoff and Özcan [14] highlighted that the marginal cement zone is among the most critical determinants of long‐term restoration success; the present case demonstrates that failure to control this zone during cementation carries measurable clinical consequences.

Clinical management of the cement excess involved mechanical removal using hand instruments, finishing burs, and rubber polishing cups, an approach consistent with published recommendations [22, 23]. Cement excess in all accessible areas, including the interproximal regions of Teeth 21 and 22, was removed, and no further signs of inflammation were detected at those sites at follow‐up. Although the complete absence of microscopic cement traces within the subgingival sulcus cannot be guaranteed following clinical removal procedures, the resolution of clinical inflammatory signs suggests that the threshold of cement accumulation sufficient to sustain a local inflammatory response was effectively addressed. This outcome underscores the clinical relevance of postcementation cement management when using insoluble resin cements. Nonetheless, the general principle remains clinical cement control; ideally during the initial gel phase before final light activation, is the most reliable strategy for preventing cement‐related complications, as it removes excess at its source before polymerization renders it tenaciously adherent [24]. At this stage, the cement has undergone sufficient initial set to be displaced as a cohesive mass without smearing further into the sulcus yet has not reached the hardness that makes mechanical removal demanding [24]. Regarding instrument selection for gel‐phase removal, the literature supports the use of a microbrush, which produces a more homogeneous and regular interfacial area compared with scalpel blade techniques; in a controlled in situ study, Brito et al. [25] demonstrated that the scalpel blade produced significantly higher mean surface roughness at the marginal interface. Increased surface roughness at the cement margin is clinically relevant because it promotes bacterial adhesion and biofilm development, potentially accelerating biodegradation of the resin cement matrix and compromising long‐term marginal integrity [25]. Regardless of the removal technique employed, postcementation polishing of the resin cement margin is recommended to further reduce surface roughness and mitigate biodegradation [25]. These considerations support the integration of a structured intraoperative cement‐control protocol; in particular the use of a microbrush at the gel phase followed by final polishing, as a definitive clinical requirement, especially when using self‐adhesive dual‐cure formulations such as RelyX U200, where the narrow working window makes timely and technique‐sensitive management essential to long‐term restoration success.

Limitations of this study include its single‐patient, descriptive design and the cross‐sectional nature of the initial SEM assessment at one time point. Cement thickness measurements were performed using ImageJ image analysis as a semiquantitative estimation based on SEM scale bar calibration; these values are indicative rather than analytically validated, and direct comparison with laboratory‐derived thresholds should be interpreted accordingly. Follow‐up periodontal documentation was limited to clinical probing without photographic records. Despite these limitations, the case provides a clinically meaningful and well‐documented illustration of a common and frequently underrecognized problem in fixed prosthodontics, and the observed periodontal improvement adds clinical support to the proposed causal mechanism.

## 4. Conclusion

This case report demonstrates that self‐adhesive resin cement excess at the cervical margins of metal‐ceramic crowns may persist subgingivally for extended periods without causing patient‐reported symptoms. Unlike conventional water‐soluble luting cements, which can partially self‐correct through gradual dissolution in oral fluids, polymerized resin cement such as RelyX U200 is insoluble and will not be displaced from the restoration margin without active mechanical intervention. SEM analysis with ImageJ‐based thickness estimation proved to be a valuable noninvasive approach for documenting the cementation interface in existing restorations. Following mechanical cement removal, resolution of bleeding on probing and reduction of probing depths were observed at all affected sites, including the interproximal regions of Teeth 21 and 22, providing clinical evidence of a causal relationship between cement excess and local gingival inflammation. Clinicians must recognize that finishing and polishing after resin cementation is a definitive clinical requirement, not a discretionary step, and that the perceived simplicity of self‐adhesive cement protocols does not reduce the demands of intraoperative cement management. Early and thorough removal of cement excess, before complete polymerization, remains the most reliable strategy for preserving long‐term periodontal health and restoration longevity.

## Funding

This study was supported by the Coordenação de Aperfeiçoamento de Pessoal de Nível Superior (10.13039/501100002322, 3110/2010).

## Conflicts of Interest

The authors declare no conflicts of interest.

## Data Availability

Data available on request from the authors.
